# Proteomic analysis of anthers from wild-type and photosensitive genetic male sterile mutant cotton (*Gossypium hirsutum* L.)

**DOI:** 10.1186/s12870-014-0390-4

**Published:** 2014-12-30

**Authors:** Ji Liu, Chaoyou Pang, Hengling Wei, Meizhen Song, Yanyan Meng, Shuli Fan, Shuxun Yu

**Affiliations:** College of Agronomy, Northwest A&F University, Yangling, 712100 Shaanxi Province China; State Key Laboratory of Cotton Biology, Institute of Cotton Research, Chinese Academy of Agricultural Sciences, Anyang, 455000 Henan Province China; Hubei Provincial Key Laboratory for Protection and Application of Special Plants in Wuling Area of China, College of Life Soiences, South Central University for Nationalities, Wuhan, 430064 Hubei Province China

## Abstract

**Background:**

Male sterility is a common phenomenon in flowering plant species, and it has been successfully developed in several crops by taking advantage of heterosis. Using space mutation breeding of upland cotton, a novel photosensitive genetic male sterile (PGMS) mutant was isolated. To take advantage of the PGMS lines in cotton hybrid breeding, it is of great importance to study the molecular mechanisms of its male sterility.

**Results:**

Delayed degradation of the PGMS anther tapetum occurred at different developmental stages as shown by analysis of anther cross-sections. To gain detailed insights into the cellular defects that occurred during PGMS pollen development, we used a differential proteomic approach to investigate the protein profiles of mutant and wild-type anthers at the tetrad, uninucleate and binucleate pollen stages. This approach identified 62 differentially expressed protein spots, including 19 associated with energy and metabolic pathways, 7 involved with pollen tube growth, 5 involved with protein metabolism, and 4 involved with pollen wall development. The remaining 27 protein spots were classified into other functional processes, such as protein folding and assembly (5 spots), and stress defense (4 spots). These differentially expressed proteins strikingly affected pollen development in the PGMS mutant anther and resulted in abnormal pollen grain formation, which may be the key reason for its male sterility.

**Conclusions:**

This work represents the first study using comparative proteomics between fertile and PGMS cotton plants to identify PGMS-related proteins. The results demonstrate the presence of a complicated metabolic network in anther development and advance our understanding of the molecular mechanisms of microgamete formation, providing insights into the molecular mechanisms of male sterility.

**Electronic supplementary material:**

The online version of this article (doi:10.1186/s12870-014-0390-4) contains supplementary material, which is available to authorized users.

## Background

Male sterility is a widespread phenomenon described in over 150 flowering plant species [1]. There are two major types of male-sterile plants, those exhibiting cytoplasmic male sterility (CMS) and those exhibiting genetic male sterility (GMS). Because of its important role in the use of hybrid vigor, there are many reports on the traits associated with male sterility, especially in rice [2-4]. CMS is a maternally inherited trait, characterized by a mitochondrial energy deficiency, CMS protein cytotoxicity and premature tapetal programmed cell death (PCD) [3]. Wild Abortive CMS (CMS­WA), a well-studied CMS line, has been exploited to produce the majority of “three­line” rice hybrids since the 1970s in China [5]. In the CMS-WA line *WA352*, a new mitochondrial gene confers the CMS-WA phenotype because its protein interacts with the nuclear-encoded mitochondrial protein COX11. WA352 accumulates preferentially in the tapetum of the anther, thereby inhibiting COX11 function in peroxide metabolism, triggering premature tapetal PCD and consequent pollen abortion [3].

Photosensitive genetic male sterile (PGMS) is a special type of GMS in which pollen fertility is regulated by day-length, and PGMS mutants are ideal female parents in hybrid production. Nongken 58S, a spontaneously occurring mutant of *japonica* rice cultivar Nongken 58, is completely sterile under long-day conditions, whereas its fertility varies from partial to full under short-day conditions [6]. Premature tapetum degeneration has been proposed to be a major reason for this variation in fertility [2,7]. The *carbon starved anther* (*csa*) mutant, another type of PGMS mutant, displays male sterility under short-day conditions but is fertile under long-day conditions. *CSA* has the key role in regulating the sugar partitioning that is required for rice anther development and pollen maturation [8]. Thus, under short-day conditions, the *csa* mutation leads to reduced assimilate allocation, resulting in male sterility. However, this mutation was partially rescued in *csa* plants under long-day conditions, as indicated by increased fertility [4]. It is of great importance to overcome the problems in the current hybrid rice systems with these studies, and the clearly understanding of CMS and GMS mechanisms in rice will greatly benefit large-scale crop breeding programs [9].

Upland cotton (*Gossypium hirsutum* L.) is an important economic crop that is used mainly for producing textile fiber. It has strong heterosis in boll number, boll weight, and seed cotton yield, and hybrid seeds are widely produced in India and China [10]. Using space mutation breeding of upland cotton, we previously isolated the novel PGMS mutant CCRI9106 (MT), which is male sterile under long-day conditions and fertile under short-day conditions and expresses a virescent marker [11]. To take advantage of the PGMS lines in hybrid breeding, it is important to study their molecular mechanisms. Transcriptome profiling analyses of anthers in MT and wild-type (WT) lines indicates that the ubiquitin-proteasome system is induced in MT uninucleate pollen (UNPs) under long-day conditions. This induction is likely to cause the degradation of pollen proteins, resulting in male sterility [11]. Whereas proteins are the main effectors of most cellular functions, there is an information gap in how the genome, the transcriptome and cellular processes are related because of posttranslational modifications, such as phosphorylation and glycosylation [12]. Thus, to better understand sterility mechanisms in cotton, it is important to conduct proteomic studies of MT and WT anthers.

Proteomics is an essential tool for elucidating gene functions and interactions. It has been widely used to reveal changes in protein expression levels between sterile and fertile anthers in several plants, and the resulting data have been used to explain plant sterility mechanisms. The application of proteomic technology has identified several proteins in rice that are correlated with male sterility and that have roles in protein synthesis, signal transduction, cell death and carbohydrate metabolism [13]. In tomato, a proteomic analysis between wild-type and *7B-1* male-sterile mutant anthers revealed that the proteasome and 5B protein, which have potential roles in tapetum degeneration, are down-regulated in the male-sterile mutant. Cystatins, regulators of endogenous proteolytic activities during seed maturation and germination and in PCD, were up-regulated in male-sterile mutants [14]. Another proteomic analysis showed that proteins associated with carbohydrate and energy metabolism, photosynthesis and flavonoid synthesis, which might also have roles in pollen development, were all down-regulated in the CMS anthers of *Brassica napus* [15]. A differential proteomic studies of the GMS line and fertile line anthers of upland cotton found that several carbohydrate metabolism- and photosynthesis-related enzymes cytosolic ascorbate peroxidase 1 and glutaminyl-tRNA synthetase at lower levels in the mutant anthers, which may play important role in pollen development [16]. Furthermore, other proteomic studies have been carried out in the *male sterile 8* anthers of *Zea mays* [17], *YX-1* male-sterile mutant anthers of wolfberry [18], and MES-induced male sterility in rapeseed [19]. To date, researches have made great progress in elucidating the mechanisms of male sterility. However, to our knowledge, no proteomic study of cotton PGMS anthers has been reported. The study of male sterile lines will advance the use of hybrid vigor in cotton.

In the present study, two-dimensional gel electrophoresis (2-DE) coupled with MALDI-TOF-MS was used to investigate differences between the protein profiles of MT and WT anthers during three key developmental stages. Sixty-six protein spots were differentially expressed, and 62 of them were successfully identified by MALDI-TOF-MS analysis. These proteins are involved in energy and metabolic pathways, protein metabolism, pollen wall development, pollen tube growth and other functional processes. The comparison of protein profiles between WT and PGMS anthers is of critical significance in understanding anther and pollen development and will provide new insight into male sterility.

## Results

### WT and MT phenotypes under long-day conditions

**Figure 1:**
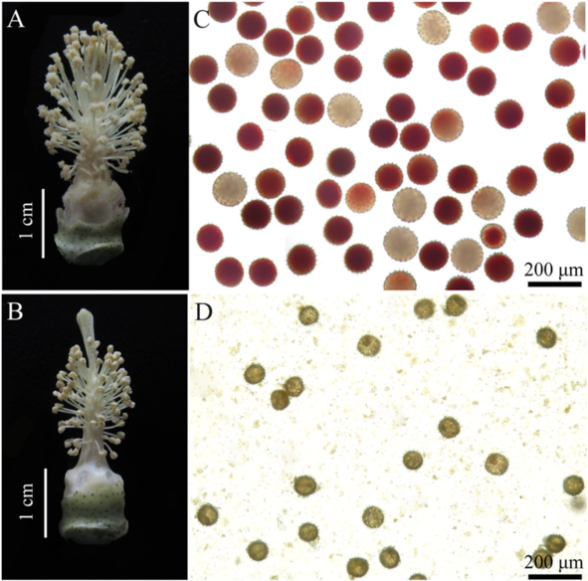
**Flower and pollen phenotypes of WT and PGMS MT cotton (** ***Gossypium hirsutum*** **L.).** The flower and pollen phenotypes are shown in **A** and **C**, respectively, for WT, and **B** and **D**, respectively, for MT.

Under the natural long-day conditions, MT and WT flowered in mid-July. Compared with the WT flower (Figure 1A), the MT flower was smaller and displayed abnormal floral phenotypes with shorter filaments and shriveled anthers (Figure 1B). Furthermore, the MT anther did not dehisce, and no visible pollen grains could be observed (Figure 1B).

To determine whether the MT could produce normal pollen grains, anthers from MT and WT were expressed using tweezers and stained with 2% TTC. Unlike WT mature pollen (Figure 1C), the MT pollen grains were aborted and could not be deeply stained by TTC, indicating that they were not viable (Figure 1D). Also, MT plants did not set seeds after self-crossing but did when cross-pollinated with the WT. Consistent with our previous study [11], genetic analysis showed that about one-quarter of the F_2_ progeny were sterile, whereas the remainder displayed normal fertility, indicating that the sterility was caused by a single recessive allele (fertile/sterile = 144:45; *χ*^2^ = 0.18 for 3:1, P < 0.05). These results suggest that the MT cannot produce viable pollen and is male sterile.

### Anther development

**Figure 2:**
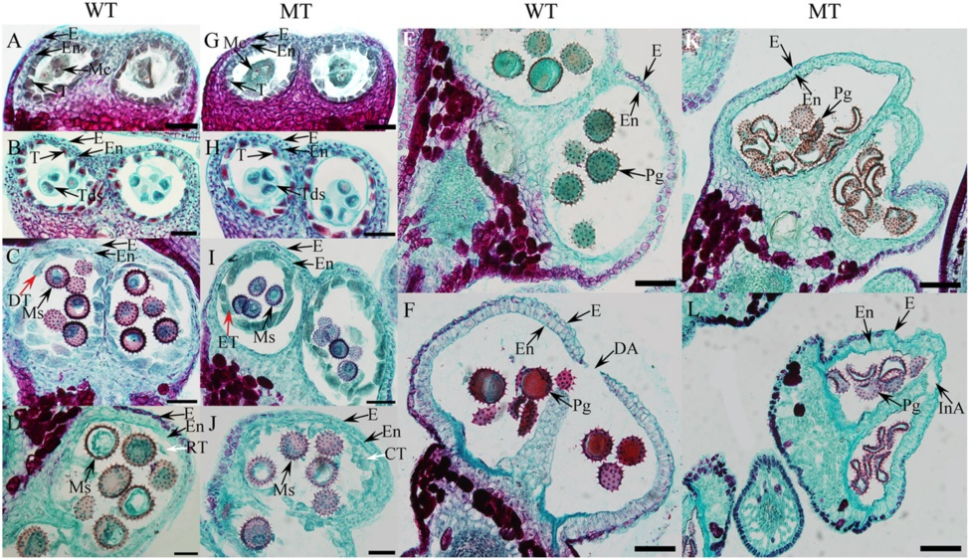
**Cross-sections of anthers from wild-type (WT) and PGMS mutant (MT) cotton (** ***Gossypium hirsutum*** **L.) at different developmental stages.** Cross-sections of WT and MT anthers at the **(A, G)** meiotic stage (MC), **(B, H)** TTP stage, **(C, I)** early UNP stage, **(D, J)** late UNP stage, **(E, K)** BNP stage and **(F, L)** flowering stage are shown. The red arrows **(C, I)** indicate the degraded tapetum in the WT and the entire tapetum in the MT. The white arrows **(D, J)** indicate the residual tapetum in the WT and condensed tapetum in the MT. Bars = 50 μm in **(A)** to **(D)** and **(G)** to **(J)**, and 100 μm in **E**, **F**, **K** and **L**. E, epidermis; En, endothecium; Mc, mother cells T, tapetum; Tds, tetrads; Ms, microspore; DT, degraded tapetum; ET, entire tapetum; RT, residual tapetum; CT, condensed tapetum; Pg, pollen grain; DA, dehisced anther; and InA, indehisced anther.

To gain more detailed insights into the cellular defects occurring during pollen development in the MT, cross-sections of anther samples from the WT and MT were examined at different developmental stages determined by flower bud length [20,21]. No cytological differences in the anther tissues prior to the tetrad stage were observed between MT and WT. The pollen mother cells (PMCs; flower bud length, ~3–4 mm) underwent meiosis (Figure 2A,G), resulting in the generation of tetrads (flower bud length, ~4.5–5 mm), surrounded by the dense tapetum (Figure 2B, H). Then, the unicellular microspores were released and enlarged in both lines. Cytological abnormalities first appeared in the MT in the tapetum cells at the early UNP stage (flower bud length, ~5–5.5 mm). During this stage, the tapetum in the WT began to degenerate (Figure 2C), thus supplying nutrients to the microspore, which is essential for microspore development. In contrast, the tapetum failed to degenerate in the MT, which appeared to have smaller microspores (Figure 2I).

The tapetum continued to degenerate, and little remained in the locule (Figure 2D) at the late UNP stage (flower bud length, ~5.5–6 mm). This was totally different from what occurred in the MT anther, in which the tapetum disappeared slowly and was still mostly present at the same stage (Figure 2J). As a consequence, during the BNP stage (flower bud length, ~10 mm for WT and 9 mm for MT), the MT pollen grains failed to accumulate storage materials, and the microspores lacked cytoplasm and were irregular in shape (Figure 2K). In contrast, the WT microspores were full of cytoplasm and were a regular round shape (Figure 2E). At flowering stage, the endothecium expanded and the anther dehisced to release mature pollen grains in the WT (Figure 2F). Consistent with the previous observations, the MT anther was shriveled and did not dehisce, resulting in an aborted pollen release (Figure 2L).

### Total sugar content measurement

**Figure 3:**
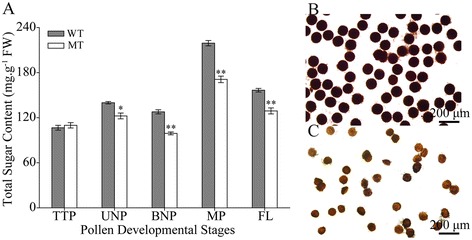
**Total soluble sugar content in anthers and starch staining of pollen grains from wild-type (WT) and PGMS mutant (MT) cotton (** ***Gossypium hirsutum*** **L.). (A)** The total soluble sugar content in different developmental stages. **(B, C)** Starch staining of **(B)** WT and **(C)** MT pollen grains. Data represent the mean and standard deviation from three replications. *P < 0.05; **P < 0.01 according to Student’s t-test. The exact values were shown in Additional file 1.

Because of no substances formed in MT pollen grains (Figure 2L) and altered protein expression patterns in the carbohydrate metabolism pathway in MT plants as compared with WT plants (Table 1), we hypothesized that the MT anthers may have defects in sugar accumulation and starch synthesis. In the WT anthers, the total soluble sugar content increased from the TTP to the UNP stage, declined during the BNP stage and significantly increased at the mature pollen stage (1 day before flowering) before decreasing again at flowering (Figure 3A, Additional file 1). Compared with the WT, there was no difference in the total soluble sugar content in the MT anther at the TTP stage; however, it was significantly lower at later developmental stages (Figure 3A, Additional file 1). Moreover, the WT pollen contained an abundant amount of starch, as indicated by dark staining with I_2_-KI (Figure 3B). In contrast, the MT pollen was only lightly stained by I_2_-KI (Figure 3C), indicating limited starch synthesis. These results suggest that the altered gene expression patterns in the carbohydrate metabolism pathway cause a reduced accumulation of total sugars and limited starch synthesis in MT anthers, which could be responsible for male sterility.

**Table 1:** **Differential protein spots between WT and MT anthers identified by MALDI-TOF-MS**

**Sp. No** ^**a**^	**Protein ID** ^**b**^	**Description** ^**c**^	**Sco.** ^**d**^	**The. MW (Kd)/pI** ^**e**^	**Cov. rate** ^**f**^	**Uniprot ID** ^**g**^	**Sim.** ^**h**^	**Average ratio** ^**i**^			**Cel. Loc.** ^**j**^	
								**TTP**	**UNP**	**BNP**	**Loc**	**RC**
**Pollen wall development**												
137	Cotton_A_15299	Chalcone synthase	129	42.81/5.37	31.77%	P30078	44.73	***0.63***	***0.43***	***NDS***	S	4
140	Cotton_D_gene_10017980	Pyruvate dehydrogenase E1 component subunit beta	196	39.51/5.89	39.50%	P52904	83.38	0.77	***0.47***	***0.42***	M	3
141	Cotton_D_gene_10001804	Pyruvate dehydrogenase E1 component subunit beta	194	36.19/4.75	28.74%	P52904	90.42	1.00	***0.48***	***0.50***	_	2
145	Cotton_A_16390	Enoyl-[acyl-carrier-protein] reductase	145	41.86/8.18	47.06%	P80030	83.6	***0.43***	***0.07***	-	C	2
**Protein metabolic**												
37	Cotton_A_32277	3-isopropylmalate dehydratase small subunit	139	27.59/6.95	27.03%	Q8YX03	44.76	***0.46***	***0.37***	***0.06***	C	1
155	Cotton_D_gene_10025384	3-isopropylmalate dehydratase small subunit	282	27.57/6.87	25.10%	Q8YX03	44.76	***1.62***	***0.30***	1.19	C	1
39	Cotton_A_02938	Proteasome subunit alpha type-2-B	208	33.39/8.57	39.07%	Q8L4A7	93.64	0.93	0.96	***1.58***	_	3
168	Cotton_A_23018	Proteasome subunit beta type-1	187	24.94/7.30	68.61%	O82531	92.83	***1.64***	***1.79***	1.07	_	3
63	Cotton_A_15704	26S protease regulatory subunit 6B	296	46.86/5.27	44.47%	Q9SEI4	95.88	***2.03***	0.86	1.46	_	3
**Pollen tube growth**												
138	Cotton_A_04015	Probable pectinesterase/pectinesterase inhibitor 58	175	35.79/5.06	24.30%	Q9FJ21	54.22	-	-	***0.47***	_	2
143	Cotton_A_28846	Probable pectinesterase/pectinesterase inhibitor 58	192	76.15/6.76	23.18%	Q9FJ21	54.22	-	-	***0.50***	_	2
103	Cotton_D_gene_10005074	Probable pectinesterase/pectinesterase inhibitor 58	225	19.43/4.62	43.43%	Q9FJ21	53.9	***0.52***	***0.20***	***0.21***	S	1
166	Cotton_D_gene_10000106	Pectinesterase PPME1	473	41.07/6.53	49.33%	Q84WM7	55.21	-	-	***NDS***	S	4
173	Cotton_D_gene_10000106	Pectinesterase PPME1	428	41.08/5.82	48.53%	Q84WM7	55.21	1.18	1.04	***0.21***	S	4
102	Cotton_A_28902	Anther-specific protein LAT52	135	19.74/4.66	43.43%	P13447	45.51	-	-	***NDS***	S	1
122	Cotton_A_35804	Anther-specific protein LAT52	169	20.25/4.75	27.78%	P13447	48.8	-	-	***NDS***	S	1
**Energy and metabolism process**												
38	Cotton_A_33732	Triosephosphate isomerase	136	27.73/5.17	46.09%	Q9SKP6	89.84	1.20	0.96	***0.03***	_	2
52	Cotton_A_22450	Malate dehydrogenase	119	35.90/6.51	32.23%	Q08062	92.15	1.24	1.02	***1.59***	_	5
164	Cotton_D_gene_10010185	Galactose oxidase	255	70.33/6.15	35.60%	P0CS93	23.61	-	-	***0.21***	S	3
165	Cotton_A_14520	Galactose oxidase	242	212.1/6.07	23.04%	I1S2N3	24.26	-	-	***0.10***	S	2
18	Cotton_A_28004	ATP synthase subunit	217	19.56/4.66	50.00%	Q9FT52	81.55	1.09	0.72	***0.65***	_	3
48	Cotton_A_03219	NADH dehydrogenase [ubiquinone] flavoprotein 2	299	28.78/7.76	31.89%	O22769	86.27	0.92	***0.66***	1.05	M	1
99	Cotton_D_gene_10017814	NADH dehydrogenase [ubiquinone] 1 alpha subcomplex subunit 5	222	19.30/4.57	40.36%	P80266	86.49	0.95	***0.61***	***0.59***	_	5
9	Cotton_D_gene_10034077	Deoxyuridine 5′-triphosphate nucleotidohydrolase	411	18.42/6.11	72.16%	Q9STG6	79.88	***1.64***	0.95	***2.24***	_	4
10	Cotton_D_gene_10034077	Deoxyuridine 5′-triphosphate nucleotidohydrolase	86.6	18.42/6.11	38.64%	Q9STG6	79.88	***1.62***	0.92	***1.88***	_	4
32	Cotton_D_gene_10028012	Succinyl-CoA ligase [ADP-forming] subunit beta	137	45.63/6.78	55.11%	Q84LB6	87.47	***1.69***	0.77	1.13	M	1
43	Cotton_A_19267	Probable 6-phosphogluconolactonase 4	72.6	35.31/6.96	24.16%	A2Z3C4	73.81	***1.54***	0.90	1.17	C	1
55	Cotton_A_33551	Caffeic acid 3-O-methyltransferase	124	40.74/5.63	26.49%	P46484	81.35	***2.00***	0.87	0.78	_	2
56	Cotton_D_gene_10035451	S-adenosylmethionine synthase	299	43.49/5.59	67.43%	Q8GTL5	97.4	1.26	1.35	***1.71***	_	2
57	Cotton_D_gene_10028579	S-adenosylmethionine synthase 1	226	43.45/5.77	56.23%	Q9AT56	96.95	1.10	1.11	***2.76***	_	2
58	Cotton_D_gene_10008718	S-adenosylmethionine synthase 2	251	43.51/5.77	60.56%	Q9AT55	97.96	0.80	0.92	***1.57***	_	2
69	Cotton_A_16872	Probable aldo-keto reductase 1	292	49.05/8.27	17.46%	C6TBN2	66.57	1.04	0.99	***1.55***	_	3
70	Cotton_A_16872	Probable aldo-keto reductase 1	113	49.05/8.27	17.69%	C6TBN2	66.57	0.93	0.90	***1.63***	_	3
74	Cotton_D_gene_10014349	Probable mannitol dehydrogenase	151	39.52/6.08	22.80%	Q9ZRF1	76.14	0.85	***1.82***	1.45	_	4
85	Cotton_D_gene_10036599	Probable cinnamyl alcohol dehydrogenase 9	96.6	39.57/5.67	22.84%	P42734	79.89	***2.24***	***2.11***	***3.47***	_	3
**Protein folding and assembly**												
20	Cotton_A_13598	23.6 kDa heat shock protein	220	23.48/5.22	55.77%	Q96331	62.15	***1.55***	***NDS***	-	M	5
59	Cotton_A_16089	Protein disulfide-isomerase	284	55.70/5.06	38.99%	Q9XF61	75.32	***1.52***	1.17	1.03	S	1
71	Cotton_A_21989	Elongation factor Tu	459	49.46/6.62	53.20%	Q9ZT91	85.93	1.11	1.48	***2.25***	C	5
91	Cotton_D_gene_10037266	17.3 kDa class II heat shock protein	111	17.56/6.02	66.67%	O82013	77.61	***1.65***	0.96	1.13	_	1
124	Cotton_A_18176	Hsp70 nucleotide exchange factor FES1	237	40.89/4.93	34.95%	Q0V4C4	36.61	0.80	0.74	***0.47***	_	3
**Stress defense**												
126	Cotton_D_gene_10025297	L-ascorbate peroxidase 1	295	44.29/6.09	41.79%	Q05431	74.49	0.97	0.93	***0.61***	_	2
100	Cotton_A_23038	Aldehyde dehydrogenase family 2 member B4	129	58.17/7.25	28.63%	Q9SU63	77.82	1.17	***0.65***	***0.60***	M	4
170	Cotton_A_23038	Aldehyde dehydrogenase family 2 member B4	191	58.17/7.25	29.57%	Q9SU63	77.82	1.11	1.45	***2.08***	M	4
171	Cotton_A_23038	Aldehyde dehydrogenase family 2 member B4	559	58.17/7.25	48.02%	Q9SU63	77.82	***0.53***	1.46	***1.52***	M	4
**Other functions**												
2	Cotton_D_gene_10029341	Glycine-rich RNA-binding protein	373	17.13/8.4	81.07%	Q03878	84.15	0.91	***1.61***	1.36	_	4
3	Cotton_D_gene_10029341	Glycine-rich RNA-binding protein	335	17.13/8.4	77.51%	Q03878	84.15	1.41	1.11	***1.62***	_	4
23	Cotton_A_35766	Pathogenesis-related protein 5	152	24.02/4.28	16.07%	P28493	63.64	-	***1.51***	1.02	C	4
31	Cotton_D_gene_10012588	Nuclear migration protein nudC	210	33.16/4.87	38.75%	O35685	61.18	0.94	1.15	***0.57***	_	5
34	Cotton_D_gene_10010187	Probable lactoylglutathione lyase	82.1	40.25/7.53	40.56%	Q8W593	78.61	***1.92***	1.08	0.86	C	3
35	Cotton_A_02073	Soluble inorganic pyrophosphatase 1	70.6	32.32/6.38	32.87%	Q9LXC9	80.07	***1.53***	0.88	0.90	C	3
44	Cotton_A_28411	Probable rhamnose biosynthetic enzyme 1	202	33.98/5.79	35.67%	Q9SYM5	82.31	1.03	0.86	***2.55***	_	3
51	Cotton_A_33743	Polygalacturonase QRT3	281	52.62/5.75	31.38%	O49432	63.52	0.97	1.12	***2.44***	C	3
83	Cotton_A_17250	Alpha-1,4-glucan-protein synthase 1	86.7	12.58/5.55	34.86%	Q9SC19	96.3	***2.92***	0.92	1.34	_	5
86	Cotton_D_gene_10037393	Profilin	233	14.41/5.48	42.86%	O49894	83.46	0.91	1.13	***0.35***	_	2
97	Cotton_A_29462	Leucine-tRNA ligase	75.1	111.1/7.00	20.18%	Q2S415	60.18	1.07	0.68	***0.66***	C	2
106	Cotton_A_35377	Putative pinene synthase	63.3	43.85/7.38	32.89%	P0CV97	47.09	-	-	***0.18***	S	4
117	Cotton_D_gene_10024340	Phosphoglycolate phosphatase	96.9	27.67/4.57	31.73%	Q5PLX6	36.47	1.05	***0.64***	***0.64***	_	3
156	Cotton_A_00485	Bifunctional monodehydroascorbate reductase and carbonic anhydrase nectarin-3	164	28.06/6.35	27.24%	Q84UV8	41.67	-	-	***0.62***	S	1
157	Cotton_A_00484	Nacrein-like protein C1	104	24.05/6.17	28.77%	A0ZSF6	54.05	-	-	***0.28***	_	2
174	Cotton_A_12497	Alpha-1,4-glucan-protein synthase	130	41.75/6.65	43.29%	P85413	93.55	1.03	0.88	***0.35***	_	3
19	Cotton_D_gene_10038772	Uncharacterized protein	71.5	10.81/4.81	57.29%			1.06	1.14	***1.97***	_	5
175	Cotton_D_gene_10017750	Uncharacterized protein	330	18.36/5.88	37.74%			0.83	***1.71***	***NDF***	_	2

^a^Spot No corresponding to spots in protein maps.

^b^Protein ID of the matched protein from the Cotton Genome Project (CGP, Institute of Cotton Research of CAAS) database (http://cgp.genomics.org.cn/page/species/index.jsp). The IDs started by “Cotton_A” were sequences from *Gossypium arboretum* genome, and “Cotton_D_gene” represent *Gossypium raimondii* genome.

^c^Description of the protein in UniProtKB.

^d^Score obtained from Mascot for each match, and the cutoff was 62.

^e^Theoretical molecular mass and isoelectric point.

^f^Coverage rate, percentage of predicated protein sequence covered by matched sequences.

^g^UniProt ID of the homolog in UniProtKB.

^h^Similarity between the identified protein and its homolog in UniProtKB.

^i^Average ratio is a ratio of the protein spots %volume ratio between the MT and WT. Ratios marked by black and italic showed p-value < 0.05 by Student’s t-test. “-”: not detectable in both maps, NDS: not detectable in the sterile MT map, NDF: not detectable in the fertile WT map.

^j^Cellular locations predicting used TargetP 1.1 Server. Loc is the locations. C, Chloroplast, M, Mitochondrion, S, Secretory pathway, “_”, Any other location. RC, Reliability Class, from 1 to 5, where 1 indicates the strongest prediction and 5 reliability classes.

### Proteomic analysis

As discussed above, the abnormality was firstly observed at UNP stage between the MT and WT and it was entirely different at BNP stage. Therefore, we collected anther samples at the TTP (flower bud length, 4.5–5 mm), UNP (flower bud length, 5.5–6 mm) and BNP (flower bud length, 10 mm for WT and 9 mm for MT) stages from MT and WT buds for proteomic analyses to evaluate pollen development. Multiple 2-DE gels of the MT and WT at the TTP, UNP and BNP stages were acquired, and the best gels were used as reference maps. A spot-to-spot comparison and quantitative image analysis revealed that 66 proteins changed significantly (P < 0.05) in relative abundance by a minimum of a 1.5-fold change (the upper and lower limits were set to 1.5 and 0.67, respectively) in at least one stage (Additional file 2).

**Figure 4:**
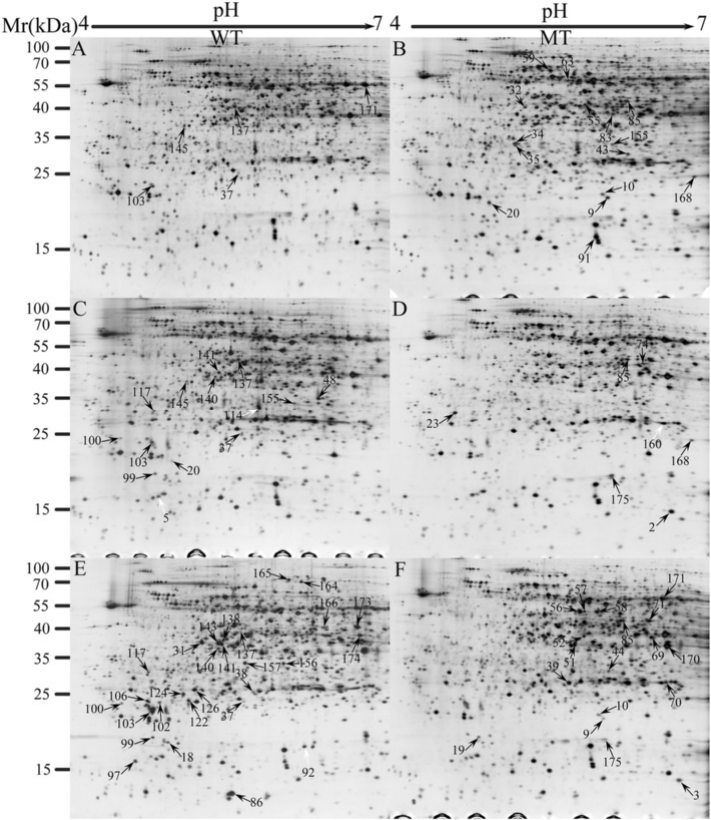
**Representative 2-DE images for total anther proteins of wild-type (WT) and PGMS mutant (MT) cotton (** ***Gossypium hirsutum*** **L.) at the TTP, UNP and BNP stages.** Silver-stained reference maps of the WT at TTP **(A)**, MT at TTP **(B)**, WT at UNP **(C)**, MT at UNP **(D)**, WT at BNP **(E)** and MT at BNP **(F)**. Each differential protein spot was marked with an arrow and number. The spots with lower intensities in the MT are showed in **(A)**, **(C)**, and **(E)**, and the spots with higher intensities are shown in **(B)**, **(D)**, and **(F)**. The white arrows indicate unidentified spots.

Among the 66 significantly changed spots, 20 were on the TTP maps, including five less-intense spots and 15 more-intense spots in the MT TTP (Figure 4A, B and Table 1). Twenty-three spots were identified on the UNP maps, including 15 less-intense spots, 1 missing spot, and 7 more-intense spots in the MT UNP (Figure 4C, D and Table 1). The greatest differences appeared between the BNP maps. A total of 46 spots were identified, including 24 less-intense spots, 17 more-intense spots, 4 missing spots, and 1 novel spot in the MT BNP (Figure 4E, F and Table 1). Of the 66 differential spots, 49 spots were found at one stage, 13 spots could be detected at two stages, and 5 spots were detected at all three stages. Furthermore, three spots (spots 102, 122 and 166) could be detected only in the WT anthers at BNP stages, suggesting the functional importance of these spots in late anther and pollen development.

### Identification and functional categorization of differentially expressed proteins

We were able to manually excise all 66 significantly different spots from the preparative Coomassie-stained 2-DE gels for further identification by MALDI-TOF-MS analysis. Sixty-two protein spots, representing 56 distinct proteins, were successfully identified by searching against our cotton_AD_nr database (Additional file 3). Some spots were identified as the same gene product. For example, two spots (2 and 3) were identified as glycine-rich RNA-binding protein, and two spots (166 and 173) were identified as pectinesterase PPME1.

The identified spots are presented in Table 1, which includes spot number, protein ID, protein names, Mascot score, coverage rate, theoretical Mass/Isoelectric Point, Swiss-Prot Protein ID, average ratio and cellular location. The subcellular localization analysis predicted that most of the proteins (34) would be localized to any other locations. Additionally, 10 localized to chloroplasts, 11 to the secretory pathway, and 7 to mitochondria (Table 1). The experimental Mr and pI predicted by SDS-PAGE has an error of about 15% compared with the theoretical value (Table 1 and Additional file 2), suggesting that some proteins appeared to be the partially degraded products of their intact proteins or post-translation modified proteins. For most of the identified proteins, there were functional annotations in the databases; however, two proteins (represented by spots 19 and 175) had no functional annotations. The annotated proteins were functionally grouped into seven categories (Table 1) by KAAS analysis according to their biological and cellular function: (1) energy and metabolic pathways, (2) pollen wall development, (3) protein metabolism, (4) pollen tube growth, (5) protein folding and assembly, (6) stress defense and (7) other functional pathways.

### Comparison with *Arabidopsis* pollen proteome

In order to get a better overview of the functionality, proteins were matched against their closest *Arabidopsis* homologues and grouped according to their predicted functions (Additional file 4). This way, most cotton protein accessions (50 of the 56 identified proteins) could be assigned to an *Arabidopsis* homologue. And only 6 accessions achieved poor matches (E-value > 10^−10^). This supports the theory that most proteins with important functions in pollen development could be detected in cotton pollen and their altered expression may result in male sterility. Furthermore, the *Arabidopsis* homologues of five spots (spots 51, 83, 137, 166, 174; Additional file 5) have been proved to affect in pollen development or pollen tube growth. The altered expression pattern of these proteins suggested that the pollen development was seriously disturbed in MT anther, responsible for the male sterility.

### Verification of differential expression via qRT-PCR

**Figure 5:**
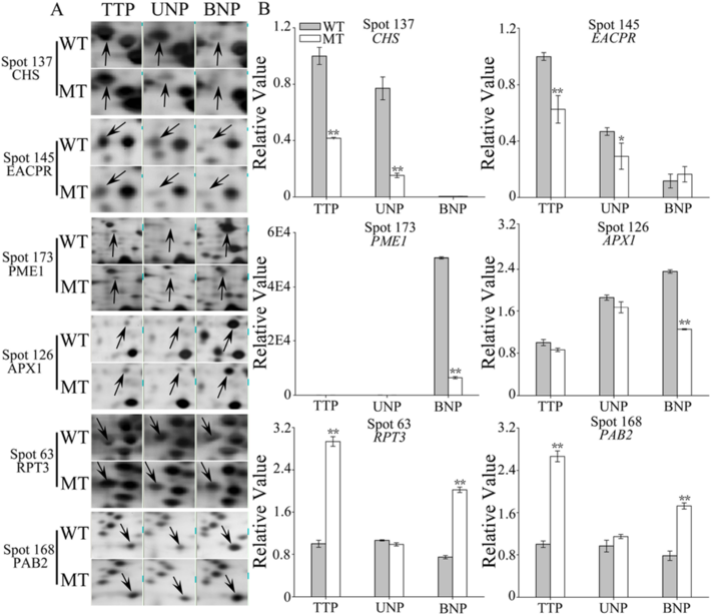
**Protein and mRNA expression levels of differentially expressed proteins spots from wild-type (WT) and PGMS mutant (MT) cotton (** ***Gossypium hirsutum*** **L.). (A)** Magnified view of six protein spots from the total proteome. **(B)** qRT-PCR analysis of the transcripts of the corresponding genes. The mRNA expression value of each gene was normalized to that of 18S rRNA, the reference gene, followed by normalization against the tetrad stage of the WT. Data represent the mean and standard deviation from three replications. *P < 0.05; **P < 0.01 according to Student’s t-test of the value at the same stage. The exact values were shown in Additional file 6. CHS (spot 137), chalcone synthase; EACPR (spot 145), enoyl-[acyl-carrier-protein] reductase; PME1 (spot 173), pectinesterase PPME1; APX1 (spot 126), l-ascorbate peroxidase 1; RPT3 (spot 63), 26S protease regulatory subunit 6B and PAB2 (spot168), proteasome subunit beta type-1.

To verify our 2-DE results and examine whether the differences in protein abundance were reflected at the transcriptional level, the mRNA expression levels of six coding genes (CHS, EACPR, PME1, APX1, RPT3 and PAB2), which corresponded to differentially expressed proteins, were analyzed by qRT-PCR. The spot intensities of CHS and EACPR were higher in the WT than in the MT at all three stages (TTP, UNP and BNP), PME1 and APX1 were higher in the WT at the BNP stage only (Figure 5A, Additional file 6). Meanwhile, the qRT-PCR results indicated that all these four genes had lower transcriptional expression level in MT anther at these stages as well (Figure 5B, Additional file 6). And both the RPT3 spot intensity and its transcription showed higher expression level in the MT at the TTP and BNP stages (Figure 5, Additional file 6). Taken together, the transcript levels of the genes encoding these five proteins demonstrated similar trends. The PAB2 protein spots showed a higher intensity in the MT at the TTP and UNP stages (Figure 5A, Additional file 6), and the mRNA levels showed a corresponding increase at the TTP stage (Figure 5B, Additional file 6).

However, the transcript levels for PAB2 at the UNP and BNP stages were inconsistent with the spot-to-spot comparison results, showing no difference at the UNP stage and an increased level at the BNP stage in the MT. In addition, APX1 had different accumulation patterns in the MT anther at the transcript and protein levels, showing an increase in protein abundance from the UNP to BNP stage but a decrease in transcript levels (Figure 5, Additional file 6). This was not surprising because numerous posttranscriptional regulatory mechanisms can cause mRNA levels to only partly correlate with protein concentrations. Moreover, it was different in transcripts and protein half-lives or translation-on-demand between mRNA and protein levels [22]. Therefore, proteomic analyses are essential for identifying the final products responsible for different cellular functions.

## Discussion

In this work, the developmental differences between the PGMS MT and WT anthers were compared by cytological and proteomic analyses. Delayed tapetum degradation was confirmed in MT anthers at the UNP stage. To acquire information on the molecular mechanisms causing these developmental differences, we further analyzed the proteomes of MT and WT anthers at the TTP, UNP and BNP stages. Sixty-two differently expressed protein spots (represent 56 distinct proteins) were successfully identified. Based on their annotated biological and cellular functions, the 56 differentially expressed proteins could participate in a range of processes during pollen development, including energy and metabolic pathways, pollen wall development, protein metabolic, pollen tube growth, and other functional proteins. These results may help us to clarify the mechanism of male sterility in PGMS mutant.

### Delayed degeneration of the tapetum in MT anthers

Formation of the anther is initiated by periclinal divisions in the hypodermal cells in the anther primordium. After mitotic divisions, the final structure consists of gametophytes surrounded by a series of cell layers, which are the tapetum, middle cell layer, endothecium and outer epidermis [23]. These layers, especially the tapetum, play important roles in pollen development, such as the production of the locular fluid and callase, and the formation of exine precursors [24]. Tapetal degeneration is induced through PCD during the late developmental stage of the anther, and premature or delayed degradation causes male sterility [25,26].

In our study, the tapetum of the WT anther started to degenerate at the early UNP stage (Figure 2C), and little remained in the locule at the BNP stage (Figure 2D). However, the tapetum failed to degenerate in the MT anthers at an appropriate stage (Figure 2I), and most still remained at late UNP stage (Figure 2J). Because of the delayed tapetum degradation in the MT anthers, no enough nutrients were available for normal microspore development. As a consequence, the MT pollen underwent abnormal development, resulting in male sterility.

### *Arabidopsis* homologues affected in pollen development

To find out how cotton pollen might differ from *Arabidopsis* pollen, we compared the proteins in our study to the proteins identified in pollen proteome of *Arabidopsis* [27-29]. Fifty of the 56 identified proteins could be assigned to an *Arabidopsis* homologue, indicating high similarity of the proteomes. The difference may result from the different samples used. In this study, the whole anther was sampled for protein extraction and identification, not the separated pollen grains as the *Arabidopsis* proteome. Chalcone synthase (CHS, spot 137) was significantly down-regulated in MT anther in all three stages (Table 1). Its *Arabidopsis* homologue AT4G34850 (LAP5 and LAP6) was absent in pollen proteome. LAP5 and LAP6 are male-organ-specific members and are expressed in anthers coincident with the timing of exine formation [30].

In *Arabidopsis*, many mutants are described that are affected in pollen development and pollen tube growth. A total of 215 genes have been surveyed by Till Ischebeck et al. [31]. From these, we found five spots to have homologues (E-value equal to or less than 10^−10^) in our study. Although the roles of these genes have not been discussed in cotton anther, the high similarity suggested conserved functions. Their changed expression pattern led to aberrant pollen development in MT anther, resulting in male sterility. However, from the 56 proteins identified, only 5 homologues have been described so far in *Arabidopsis* mutant studies, leaving tremendous room for future pollen research.

### Energy and metabolism processes

In higher plants, the development of the male gametophyte is a well-programmed and elaborate process [32], which may require more genes expression. For example, compared with other organs, more than 20,000 genes have been detected as expressing in cotton anthers [33]. To accomplish this complex process, numerous proteins are associated with energy and metabolism in anther development. It has been well studied in *Arabidopsis thaliana*. For example, of the proteins identified on the 2-DE reference proteome maps for mature pollen of *Arabidopsis thaliana*, ~40% are predicted to function in metabolism and energy generation [27,28]. Except for these 2-DE based proteomics analysis, the metabolism and energy functional categories were also overrepresented in a shotgun proteomics of *Arabidopsis* pollen [29]. Moreover, the tobacco proteome analysis from early to late pollen development demonstrated that proteins involved in primary metabolism and starch synthesis, which were required for pollen tube growth [31]. These suggested that energy and metabolism processes were the most primary processes in pollen. Disordered expression of proteins in these processes may cause male sterility [16,19]. In this study, ~31% (19) of the 62 spots identified were implicated in energy and metabolism (Table 1). Their up- or down-regulation may cause abnormal development of the MT anthers.

In detail, two of the proteins identified here had functions in carbohydrate metabolism (spot 38 representing triosephosphate isomerase, and spots 164 and 165 representing galactose oxidase), two in energy generation (spot 18 representing ATP synthase, and spots 48 and 99 representing NADH dehydrogenase) and the others were involved in metabolism processes. As the highest sink, anthers need to obtain large amounts of sugars to support their early development, and at later stages pollen maturation requires the accumulation of starch, which functions as an energy reserve for germination, thus serving as a marker of pollen maturity [8]. It has been shown that changes in expression of carbon and energy metabolism genes led to total soluble sugar content decrease at the meiosis and UNP stages in the cotton GMS mutant anthers [33]. In this study, because of the altered expression levels of carbohydrate metabolism-related genes, there was a strikingly reduced accumulation of sugars in the MT anthers at the late developmental stages (Figure 3A) and limited starch synthesis in the MT mature pollen grains (Figure 3C). As expected, the WT mature pollen grains (Figure 2F) stored a number of substances (e.g., polysaccharides, proteins, lipids and hormones) that place a high demand on energy and carbon reserves for successful germination and tube growth [19]. But the MT pollen grains were nearly empty from late UNP stage (Figure 2J). During anther development, there is an increased demand for respiratory function and cellular energy in the form of ATP. Defective in ATP synthesis may result in abnormal anther development with non-functional pollens [16,34]. In this work, two proteins in energy generation (spot 18 representing ATP synthase, and spots 48 and 99 representing NADH dehydrogenase) were significantly reduced in MT anthers, suggesting that the MT anthers were in an energy starved state.

These results suggest that the disordered gene expression in carbohydrate metabolism and energy germination resulted in reduced accumulation of total sugars, a lack of starch and other substances synthesis in the MT pollen grains, thus providing critical information augmenting our understanding of male sterility.

### Pollen wall development

The pollen wall is formed of a number of layers, the outer exine, the outer sculptured layer or sexine and the inner nexine. The exine layer is formed principally of sporopollenin, which is synthesized predominantly by the tapetum and is an aliphatic polymer comprised of a series of polymers derived from long-chain fatty acids, phenylpropanoids and oxygenated aromatic rings [24]. Its primary roles are to provide structural and physical support to the microspore cytoplasm and protection from harsh conditions, such as prolonged desiccation, high temperatures and ultraviolet light. It also facilitates pollination by attracting vectors that prefer an elaborate pollen outer wall [24]. In *Arabidopsis*, defects in sporopollenin formation can cause male sterility [23]. In this study, four proteins, represented by spots 137, 140, 141 and 145, that are involved in sporopollenin formation were found to be differentially expressed in the MT anthers (Figure 4).

Chalcone synthase (CHS, spot 137), which is the first committed enzyme in the biosynthesis of all flavonoids and is essential for pollen development and the biosynthesis of sporopollenin [35], was significantly reduced in all three stages of MT anther development. In *Arabidopsis*, *LAP5* and *LAP6* encode anther-specific proteins with homology to CHS and may play a role in the synthesis of pollen fatty acids and phenolics found in exine. Mutations in either gene result in abnormal exine patterning, whereas the *lap5 lap6* double mutant produces pollen grains devoid of exine, causing strong male sterility [30].

The proteins represented by spots 140, 141 and 145, which were classified into the fatty acid synthesis pathway, were significantly reduced as well in the MT. Fatty acids are important components of sporopollenin. Mutations that in fatty acid synthesis can cause impaired pollen wall formation [36]. Enoyl-[acyl-carrier-protein] reductase (EACPR, spot 145) is a subunit of the fatty acid synthase complex that catalyzes de novo synthesis of fatty acids. A reduced-function mutation of this gene in *Arabidopsis*, *mosaic death1* (*mod1*), causes a marked decrease in its enzymatic activity, impairing fatty acid biosynthesis and decreasing the amount of total lipids [37].

The pyruvate dehydrogenase E1 component subunit beta (PDH E1-B, spots 140 and 141) is essential for the synthesis of sporopollenin precursors. Acetyl-CoA is formed from pyruvate through a PDH complex in mitochondria, and the released acetyl-CoA is a substrate for de novo fatty acid synthesis in plastids [24]. Antisense inhibition of PDH_E1α-1 in the anther tapetum is sufficient to cause male sterility, a phenocopy of the sugar beet CMS [38]. The relatively reduced amount of sporopollenin formation–related proteins in the MT anthers could contribute to male sterility. Because fatty acids are the likely components of sporopollenin, which contributes to the formation of the protective pollen coat [24], reduced amounts of these proteins may lead to abnormal pollen coat formation in the MT. The MT pollen was irregularly shaped (Figure 1K), which may have resulted from abnormal sporopollenin formation. To uncover the detailed changes, the structure of the pollen wall will be further studied at high resolution microscope.

### Protein metabolism

As a non-photosynthetic male reproductive organ, the anther needs to obtain nutrients from source organs to support pollen development and maturation, and proteins, as well as amino acids, are important components of pollen cytoplasm [11,39]. Proteasomes are important proteases in eukaryotes and regulate many cellular processes, including metabolism, cell cycle and the proteolysis of regulatory proteins. The altered expression levels of proteasome-related enzymes in the tomato *7B-1* anthers may affect meiosis in the microspore mother cells [14]. In our previous research [11], several genes related to the ubiquitin-proteasome system were up-regulated in the MT anther at the UNP stage. Thus, under long-day conditions, the ubiquitin-proteasome system is induced in the MT at the UNP stage and likely leads to protein degradation. With insufficient protein and amino acid levels, the cytoplasm of MT pollen grains is likely to break down gradually, and the pollen grains are likely to lose activity, resulting in male sterility [11].

In this study, the proteolytic enzymes proteasome subunit α type-2-B (spot 39), proteasome subunit beta type-1 (spot 168) and 26S protease subunit 6B homolog (spot 63) (Figure 4), were up-regulated in the MT and one amino acid biosynthesis–related protein, 3-isopropylmalate dehydratase small subunit (spots 37 and 155), was down-regulated in the MT anthers. These changes may cause reduced protein and amino acid levels in MT pollen grains, although the exact mechanism for this effect is still unclear. Consistent with the previous study, the induced degradation of cytoplasmic proteins in the pollen of the MT may be another reason for its male sterility.

### Pollen tube growth

Pollen germination, along with pollen tube growth, is an essential process in the reproduction of flowering plants. The wall of the pollen tube is composed of a single layer of pectin, and pectin methylesterases (PMEs) likely play a central role in pollen tube growth and the determination of pollen tube morphology [40]. The function of PMEs in pollen tube growth and pollen germination has been well studied in several plant species. *AtPPME1* is a pollen-specific gene, and its protein is found only in the mature pollen grains and growing pollen tubes. After germinating and being cultured in vitro, pollen tubes of *atppme1* mutant pollen grains have a curved, irregular morphology and are dramatically stunted [41].

In plants, PME activities are regulated by either differential expression or posttranslational modification by specific PME inhibitor proteins (PMEIs) [42]. It has been suggested that AtPMEI2 accumulates exclusively at the pollen tube apex and regulates pollen tube wall stability by locally inhibiting PME activity [43]. Additionally, the ectopic expression of a *BoPMEI1* antisense gene in *Arabidopsis* suppresses expression of its orthologous gene, *At1g10770*, resulting in pollen tube growth retardation, partial male sterility, and reduced seed set [44].

LAT52 is also essential for pollen development, because pollen grains that express antisense *LAT52* RNA hydrate and germinate abnormally and cannot achieve fertilization [45]. Interestingly, all the three related proteins in this analysis (represented by spots 166 and 173 for the PME; spots 103, 138 and 143 for the PMEI and spots 102 and 122 for LAT52) had lower expression levels in our MT anther maps and extremely high expression levels in the WT maps, especially at the BNP stage (Figure 4). We believe that this change may reduce the accumulation of pollen components for pollen tube growth, which leads to pollen that could not germinate after maturation, resulting in nonviable pollen grains.

### Other functional proteins

The 27 remaining proteins could be classified into other diverse functional categories (Table 1). They have important roles in anther development as well, including five proteins with roles related to protein folding and assembly. The 23.6-kDa heat shock protein (HSP; spot 20), 17.3-kDa HSP (spot 91), protein disulfide-isomerase (spot 59) and elongation factor Tu (spot 71) were up-regulated in MT anthers; however, HSP70 (spot 124) was down-regulated. These proteins have been well studied and are responsible for protein folding and assembly [46]. In this study, the different expression levels of HSPs implied that protein folding and assembly are altered in MT anthers, suggesting variations in protein translation and post-translational modifications, which might lead to aberrant anther development.

Stress defense–related proteins formed another functional category that included L-ascorbate peroxidase (APX; spots 126, Figure 4) and aldehyde dehydrogenase (ALDH; spots 100, 170 and 171), which were down-regulated in the MT. These proteins are important in detoxifying reactive oxygen species damage during anther development in upland cotton [21]. Thus, the differential expression of these proteins in MT anthers may unbalance the oxidation-reduction process, which may play an important role in anther and pollen development [18,21]. In addition, other proteins with significantly altered expression levels may also influence anther and pollen development. Further studies are required to investigate the functions of these proteins.

## Conclusions

Male sterility is a common phenomenon in flowering plant species. Using space mutational breeding, a novel PGMS mutant line was developed and identified. The anthers in the MT plants underwent delayed tapetum degradation. To better understand the cellular defects that occurred during pollen development in the MT, a comparative proteomic approach was conducted, and 62 differentially expressed protein spots were identified between the PGMS MT and WT anthers at three developmental stages. These proteins were involved in energy and metabolic pathways, protein metabolism, pollen wall development, pollen tube growth and other functional processes. The differential expression of these proteins may strongly disturb pollen development in the MT anther and cause abnormal pollen grain formation, which may be the key reason for the sterility.

Delayed tapetum degradation may result in insufficient nutrition supplying for microspore maturation. As a consequence, the pollen wall underwent abnormal development and failed in accumulation of pollen components for pollen tube growth. Finally, nonviable pollen grains were formed in the MT anther. Our results may be relevant for many biological processes in anther and pollen development and provide insight into the mechanisms behind photosensitive male sterility in higher plants.

## Methods

### Plant growth and anther collection

Two *G. hirsutum* L. genotypes, a PGMS mutant CCRI9106 and its WT line, CCRI040029, were used in this study. CCRI040029 was an elite upland variety bred in our lab, and the mutant line, CCRI9106, was identified by space mutation in 2010 [11]. They were grown in an agronomic field in Anyang, Henan, P. R. China from April to October. Thirty lines (8 m in length × 0.8 m in width) were prepared for each genotype, and every 10 lines formed one replicate.

As in our previous study [21], during the anthesis period, flower buds of different lengths were observed to identify the pollen developmental stages and then were sampled for anther collection every other day. Pollen grains from each flower bud were expelled, dissolved in mixed acids (chromic acid/nitric acid/hydrochloric acid, 15/10/5, v/v/v) and then stained by 2% iodine-potassium iodide (I_2_-KI) or 2% 2,3,5-triphenyltetrazolium chloride (TTC). They were then photographed using an Olympus DP72 light microscope. To observe cross-sections, anthers were fixed in formalin-aceto-alcohol (FAA) and dehydrated in an ethanol series. The samples were then embedded in paraffin. Longitudinal sections were cut using a Leica RM2265 ultramicrotome, stained using safranin with a fast green counterstain and photographed using the Olympus DP72 light microscope.

Additionally, anthers from both MT and WT were collected during the tetrad pollen (TTP) period, as well as from the early and late UNP, binucleate pollen (BNP), mature pollen and flowering periods for further analysis. The collected anthers were immediately fixed in FAA for cross-sectioning or frozen in liquid nitrogen and stored at −80°C until proteins, total sugar and mRNA extractions were performed.

### Protein extraction and quantification

TCA–acetone method was selected for anther protein extraction [47]. Protein extractions were performed according to Pang et al. with minor modifications [48]. In brief, ~1.5 g of frozen anther was ground with 10% polyvinyl polypyrrolidone (w/w) in liquid nitrogen using a mortar and pestle. The resulting fine powder was mixed with 10% (w/v) trichloroacetic acid in cold acetone containing 0.07% (w/v) 2-mercaptoethanol for at least 2 h and subsequently centrifuged at 12,000 g for 1 h at 4°C. The pellet was washed first with cold acetone containing 0.07% (w/v) 2-mercaptoethanol and then with 80% cold acetone and finally was suspended in lysis buffer (7 M urea, 2 M thiourea, 4% CHAPS, 20 mM dithiothreitol, 2% EDTA-free protease-inhibitor). The supernatant was centrifuged at 120,000 g for 90 min at 4°C and used for further assays. The concentration of the protein solution was determined with the 2-D Quant Kit (GE Healthcare) with bovine serum albumin as a standard. The supernatants were stored at −80°C until required.

### Two-dimensional gel electrophoresis

Two-dimensional gel electrophoresis (2-DE) was performed as follows. Two technical and three biological replicates were prepared for each stage (i.e., at least six gels for each sample). Total anther proteins, 150 μg or 1.5 mg, were visualized in silver- or Coomassie-stained (Coomassie Brilliant Blue R-350; GE Healthcare) gels, respectively. Isoelectric focusing was performed with the IPGphor system (GE Healthcare). Immobiline pH 4 to 7 and 24-cm linear DryStrips (GE Healthcare) were run at 30 V for 8 h, 50 V for 4 h, 100 V for 1 h, 300 V for 1 h, 500 V for 1 h, 1000 V for 1 h and 8000 V for 12 h using rehydration buffer (8 M urea, 2% CHAPS, 20 mM DTT) containing 0.5% (v/v) IPG Buffer (GE Healthcare). The second-dimension SDS-PAGE was performed using 12.5% polyacrylamide gels without a stacking gel in an Ettan DALTsix Electrophoresis Unit 230 (GE Healthcare). For silver-staining, gels were stained with 0.25% (w/v) silver nitrate and visualized by 0.004% (v/v) formaldehyde in 2.6% (w/v) sodium carbonate. For CBB staining, gels were stained with 0.04% (w/v) PhastGel Blue R (Coomassie Brilliant Blue R-350; GE Healthcare) in 10% acetic acid and destained with 10% acetic acid. Silver-stained gels were immediately scanned at a resolution of 300 dots per inch using a PowerLook 2100XL (UMAX) and analyzed using ImageMaster platinum 6.0. The relative volume (% volume) was used to quantify and compare the spots. Spots with significant changes, at least 1.5-fold up- or down-regulated at P < 0.05 (Additional file 1), were manually excised from the CBB-stained gels.

### MALDI-TOF-MS and database searching

Excised protein spots were analyzed using a Bruker UltrafleXtreme MALDI-TOF/TOF mass spectrometer. Monoisotopic peak masses were acquired in a mass range of 500 to 3,500 Da. Five of the most intense ion signals were selected as precursors for MS/MS acquisition. Based on the peptide mass fingerprinting results and the MALDI-TOF/TOF-MS analysis, sequence similarity searches for protein identification were performed with Mascot 2.3.02 software (Matrix Science, Boston, MA, USA) using default parameters against our cotton_AD_nr database. This database includes 38,460 sequences from the *Gossypium raimondii* genome [49] and 43,097 from the *Gossypium arboreum* genome [50], the putative contributors of the D and A subgenomes, respectively, of the *G. hirsutum* AD genome. If there was no significant match, then the spot was searched against the UniProt viridiplantae database (http://www.uniprot.org/, Release 2012_12), and the highest-scoring protein was reported. The search variables were set as follows: one missed trypsin cleavage, carbamidomethyl cysteine residues as a fixed modification, methionine oxidation as a variable modification, a peptide mass tolerance of 100 ppm and a fragment ion mass tolerance of 0.4 Da.

### Protein functional classifications

The differentially expressed proteins were functionally categorized by the KEGG Automatic Annotation Server (KAAS, http://www.genome.jp/tools/kaas/) using the default parameters [51]. Then, they were classified into different categories according to their predicted biological functions. Their subcellular localizations were predicted using the TargetP 1.1 Server (TargetP, http://www.cbs.dtu.dk/services/TargetP/) with the default settings [52]. To compare with *Arabidopsis* pollen proteome (3517 proteins from *Arabidopsis* pollen proteome) [27-29], all proteins in this study were blasted for the closest *Arabidopsis* homologue with E-value ≤ 10^−10^.

### Quantitative real-time PCR (qRT-PCR)

Total RNA from anther samples was extracted using the pBiozol Total RNA Extraction Reagent (BioFlux) according to the manufacturer’s protocol. Reverse transcription reactions were performed with SuperScriptIII reverse transcriptase (Invitrogen, USA) following its protocol. Reactions were carried out using SYBR Green PCR Master Mix (Roche Applied Science, Germany) on an ABI 7500 real-time PCR system (Applied Biosystems, USA) with three replicates. Reaction volumes were 25 μL and contained 12.5 μL SYBR Green PCR Master Mix, 9.5 μL deionized H_2_O, 1 μL primers and 2 μL cDNA. Amplification reactions were initiated with a pre-denaturing step (95°C for 10 min), followed by denaturing (95°C for 10 s), annealing (60°C for 35 s) and extension (72°C for 35 s) for 40 cycles. Data were processed using the 2^-△△Ct^ method, and the 18S rRNA was used as an endogenous reference gene for data normalization, followed by normalization against the TTP of WT. The primer pairs used for qRT-PCR were designed based on the expressed sequence tag sequences from our anther cDNA library [21]. The identified protein sequences were blasted against the cDNA library, and the best hit was selected for primer design and qRT-PCR. The corresponding sequences and primers are shown in Additional file 7.

### Total sugar content measurement

Anthers were harvested and frozen at −80°C. The samples were ground into fine powder in liquid nitrogen using a mortar and pestle. Twenty milliliters of water was added to the glass tubes containing 1 g of ground anther tissue. The tubes were incubated at 100°C for 10 min and then centrifuged at 2,500 g for 5 min. A 2 mL solution containing glucose, fructose or galactose was prepared. A 200 μg · mL^−1^ glucose solution was used as the standard for optimization. An anthrone colorimetric method was adopted to determine the total sugar content in the WT and male sterile MT anthers [33].

### Availability of supporting data

The data sets supporting the results of this article are included within the article and its additional files.

## Additional files

Additional file 1:
**Total soluble sugar content in anthers of wild-type (WT) and PGMS mutant (MT) cotton (** ***Gossypium hirsutum*** **L.).**
Additional file 2:
**Relative volume (% volume) and ratio of significantly changed spots between wild-type (WT).**
Additional file 3:
**Sequences of identified proteins by MALDI-TOF-MS.**
Additional file 4:
**Comparison of cotton anther and** ***Arabidopsis*** **pollen proteins.**
Additional file 5:
**Closest** ***Arabidopsis*** **genes affected in pollen development or pollen tube growth.**
Additional file 6:
**Comparision of protein and mRNA expression levels of differentially expressed proteins spots from wild-type (WT) and PGMS mutant (MT) cotton (** ***Gossypium hirsutum*** **L.).**
Additional file 7:
**Primers and EST-sequences used for real-time PCR analysis.**

## Abbreviations

2-DE: Two-dimensional Gel Electrophor
BNP: Binucleate Pollen
CMS: Cytoplasmic Male Sterility
CSA: Carbon Starved Anther
GMS: Genetic Male Sterility
MALDI-TOF-MS: Matrix-Assisted Laser Desorption/Ionization Time of Flight Mass Spectrometry
PCD: Programmed Cell Death
PGMS: Photosensitive Genetic Male Sterile
PMC: Pollen Mother Cell
qRT-PCR: Quantitative Real-time PCR
TCA: Trichloroacetic Acid
TTC: 2,3,5-triphenyltetrazolium Chloride
TTP: Tetrad Pollen
UNP: Uninucleate Pollen
